# Automated air plasma-assisted functionalization of graphite electrodes for enhanced electrochemical sensing of uric acid

**DOI:** 10.1007/s00604-026-07939-2

**Published:** 2026-02-28

**Authors:** Mariana C. Marra, Marina Di-Oliveira, Raquel G. Rocha, Teodoro R. Terra, Robert D. Crapnell, Craig E. Banks, Eduardo M. Richter, Rodrigo A. A. Muñoz

**Affiliations:** 1 Institute of Chemistry, Federal University of Uberlândia, Uberlândia, 38408-100 Brazil; 2 Faculty of Science and Engineering, Manchester Metropolitan University, Dalton Building, Chester Street, Manchester, M1 5GD Great Britain

**Keywords:** Automated system, Graphite paper, Graphite sheet electrodes, Differential pulse voltammetry, Arc discharge plasma, Biomarker, Tesla coil

## Abstract

A lab-made robotic system is presented that integrates an arc discharge air plasma generator with automated X- and Y-axis motion, enabling rapid treatment (25 s per 0.19 cm²) of graphite sheet (GS) electrodes with improved reproducibility compared to manual handheld plasma treatment. Scanning electron microscopy (SEM) revealed an increase in surface roughness and the formation of 3D nanoflake structures following plasma treatment. Complementary Raman spectroscopy analysis showed an elevated D-to-G band intensity ratio (I_D_/I_G_), indicating a higher density of structural defects. Electrochemical characterization using cyclic voltammetry demonstrated enhanced electrochemical responses for ferricyanide and various organic compounds, including catechol, codeine, ketamine, paracetamol, sibutramine, and uric acid. The improvements were observed as increased current intensities and negative shifts in redox peak potentials, reflecting enhanced electron transfer kinetics at the treated electrode surface. The lower charge transfer resistance measured by electron impedance spectroscopy (EIS) and the higher heterogeneous electron transfer rate constant (*k*^*0*^) obtained for plasma-treated GS electrodes are consistent with these results. As proof of concept, treated GS sensors were used to detect uric acid, an important clinical biomarker, in spiked synthetic saliva and urine samples, using optimized differential pulse voltammetry (DPV) conditions. Linear ranges (between 1.0 and 400.0 and 400.0–1000.0 µmol L^− 1^) and a limit of detection (LOD) of 0.08 µmol L^− 1^ were achieved. Appropriate recovery values (106–109%) were also obtained for the analysis of spiked samples.

**Graphical abstract:**

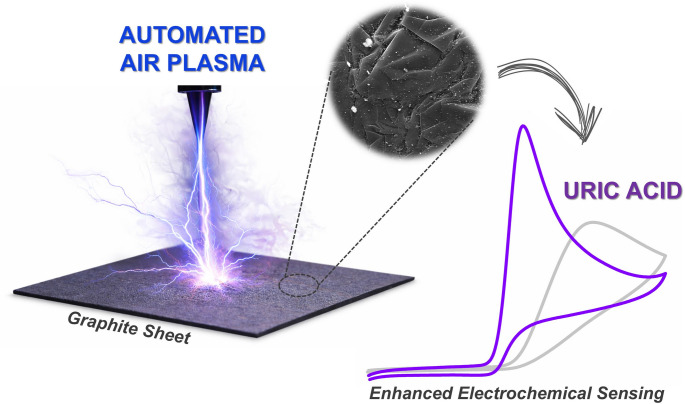

**Supplementary Information:**

The online version contains supplementary material available at 10.1007/s00604-026-07939-2.

## Introduction

 Analytical chemistry plays a fundamental role in scientific and technological progress by enabling both qualitative and quantitative analysis of chemical substances across diverse fields. Reproducibility and repeatability have long been critical concerns in analytical chemistry, as the credibility of scientific results depends on the ability to replicate experimental results [1, 2]. However, many analytical methods still require manual optimization and are highly operator-dependent, which poses significant challenges to achieving reproducibility [3, 4].

In this way, robotic systems significantly improve operational efficiency and experimental throughput, as demonstrated by recent advancements in the field [5–7]. These platforms also provide superior precision and accuracy by eliminating human intervention, ensuring consistent experimental conditions, and replacing error-prone manual procedures [6, 8]. Robots can handle a wide range of sample-processing tasks [9, 10], including applications within the electrochemistry field [11, 12]. Specifically, in electrochemical research, robotic systems have been successfully applied to a variety of experiments, including pH-dependent molecular electrocatalysis [13], energy storage [14, 15], electroanalytical sensing [16], and electrosynthesis [17].

The selection of the electrode composition is an important step for achieving optimal performance and selectivity in the development of electroanalytical devices. The electrode material plays a crucial role in influencing electron transfer kinetics, often determining the overall effectiveness of the sensor [18, 19]. In recent years, paper-based electrochemical devices (ePADs) have gained increasing attention for a wide range of applications, particularly in electrochemical sensing [18, 20].

Within this context, graphite sheets (GSs), derived from natural graphite flakes through an intercalation process, have emerged as highly attractive materials for constructing (bio)sensors. Recent studies have highlighted the growing relevance of graphite-based electrochemical sensing, demonstrating their suitability for sensitive, low-cost, and flexible sensing platforms [21–23].

This is due to a combination of advantageous properties, including excellent corrosion resistance, lower cost compared to conventional electrodes, high chemical stability, and mechanical flexibility, features that are particularly appealing for the development of wearable sensing technologies [24, 25]. Several studies have demonstrated the potential of GSs in developing electroanalytical methods for the detection of various analytes; however, a common limitation remains the requirement for electrode surface modification to improve their electrochemical performance [26–29].

Plasma treatment has emerged as a convenient and efficient approach for surface modification of carbon- and graphite-based electrodes, owing to its ability to introduce oxygen-containing functional groups, enhance surface roughness, and improve electron transfer kinetics without the use of chemical reagents [30, 31]. Techniques such as cold atmospheric plasma, dielectric barrier discharge, and arc-discharge plasma have been widely applied in electrochemical systems, biosensors, and energy-related applications due to their simplicity, scalability, and environmental friendliness.

Recently, our research group demonstrated that handheld pen-type plasma treatment can enhance the electrochemical activity of GSs [32]. This method generates free radicals through electron discharge in the presence of atmospheric gases, disrupting the bonds between sp²-hybridized carbon atoms on the electrode surface. As a result, various polar functional groups are introduced, leading to increased surface energy, enhanced hydrophilicity, and greater surface roughness [33–35]. Although this approach showed excellent results for the simultaneous detection of paracetamol and ciprofloxacin in water samples, it remains highly operator-dependent, which limits reproducibility and scalability, making it less suitable for large-scale (bio)sensor production. These limitations highlight the need for automated plasma systems with improved control and repeatability, motivating the development of the automated arc-discharge platform proposed in this work.

Uric acid (UA) is a key biomarker found in various biological fluids and plays a critical role in human physiological and metabolic processes. For example, UA concentrations in urine and serum are widely used as diagnostic indicators for several medical conditions, including hyperuricemia, gout, and Lesch–Nyhan syndrome [36, 37]. Furthermore, a previous study has suggested that abnormal UA levels may also be associated with diseases such as leukemia and pneumonia [38]. In healthy adults, UA concentrations typically range from 0.13 to 0.46 mmol L^− 1^ in blood serum and from 1.49 to 4.46 mmol L^− 1^ in urine samples [39]. Therefore, analytical platforms designed for UA detection must be capable of operating reliably within micromolar-to-millimolar concentration ranges.

Currently, the standard method for UA detection involves enzymatic assays using uricase, which catalyzes the oxidation of UA into allantoin, with hydrogen peroxide produced as a byproduct. The produced H₂O₂ is then electrochemically detected via a peroxidase enzyme. Although this uricase–peroxidase enzymatic system offers high sensitivity and selectivity, it is vulnerable to degradation under environmental conditions such as variations in pH, temperature, light, and oxygen exposure [40]. This sensitivity compromises the stability and reproducibility of the detection process [41].

In this work, we demonstrate that a lab-made robotic system offers a simple, reproducible, and rapid (~ 130 s per cm² area) arc discharge plasma treatment using atmospheric air for GS electrode surfaces. The plasma-treated GS electrodes were successfully applied for the detection of uric acid in synthetic saliva and urine samples. Compared to existing plasma treatment methods, the proposed robotic platform is more cost-effective and significantly reduces operator-dependent variability. Owing to its scalability and low cost, this approach, our study introduces an innovative exfoliation technique for producing few-layer graphene flakes, with potential applications in sensor development.

## Experimental

### Chemicals and samples

All aqueous solutions were prepared using deionized water with resistivity not less than 18 MΩ cm (Millipore Direct-Q3 water purification, MA, USA). Potassium ferricyanide (99% w/w), sodium nitrite (98% w/w), sodium chloride (99% w/w), paracetamol (98% w/w), monopotassium phosphate (98% w/w), and sodium hydrogen carbonate (99.5% w/w) were acquired from Labsynth (Diadema, Brazil). Urea (99% w/w) and uric acid (≥ 99% w/w) were obtained from Sigma Aldrich (St Louis, USA). Potassium chloride (≥ 99.5% w/w) was purchased from Êxodo Científica (São Paulo, Brazil). Sodium phosphate dibasic (99% w/w), citric acid (≥ 98% w/w), sodium sulfate (99% w/w), potassium thiocyanate (99% w/w), acetic acid (99% w/v) and phosphoric acid (85% w/v) were obtained from Vetec (Rio de Janeiro, Brazil). Boric acid (99% w/w) was obtained from AppliChem Panreac (Barcelona, Spain). Sodium hydroxide (98% w/w) was obtained from ChemiFlex (São Bernardo do Campo, Brazil). Magnesium sulfate (97% w/w) and ammonium chloride (99% w/w) were acquired from Cinética (São Paulo, Brazil). All reagents were analytical grade and employed without further purification.

A mixture of phosphoric, acetic, and boric acids (each at 0.04 mol L^− 1^) was employed for preparing Britton-Robinson (BR) buffer solutions, with pH adjusted using 1 mol L⁻¹ NaOH. These solutions were used as supporting electrolytes in electrochemical measurements.

Synthetic saliva was prepared following a procedure adapted from the literature [42], containing 0.33 g L⁻¹ potassium thiocyanate, 0.26 g L⁻¹ sodium phosphate dibasic, 1.30 g L⁻¹ urea, 0.70 g L⁻¹ sodium chloride, and 1.20 g L⁻¹ potassium chloride. Synthetic urine was prepared as described in the literature [43] using 0.37 g L⁻¹ ascorbic acid, 0.04 g L⁻¹ calcium chloride, 2.10 g L⁻¹ sodium hydrogen carbonate, 0.07 g L⁻¹ uric acid, 9.99 g L⁻¹ urea, 0.40 g L⁻¹ citric acid, 1.41 g L⁻¹ sodium sulfate, 5.20 g L⁻¹ sodium chloride, 0.95 g L⁻¹ magnesium sulfate, 0.95 g L⁻¹ monopotassium phosphate, and 1.30 g L⁻¹ ammonium chloride. Both synthetic saliva and urine samples were spiked with uric acid at two concentration levels (10 and 50 µmol L⁻¹) and subsequently analyzed after simple dilution in a supporting electrolyte, with dilution factors of 20-fold for synthetic saliva and 40-fold for urine samples.

### Electrochemical measurements

All electrochemical measurements, including cyclic voltammetry (CV), differential pulse voltammetry (DPV), and electrochemical impedance spectroscopy (EIS), were carried out using a PGSTAT204N potentiostat/galvanostat (Metrohm Autolab BV, Utrecht, The Netherlands). Data acquisition and processing (baseline correction for DPV scans) were performed using NOVA 2.1.7 software. Pyrolytic GS obtained from Panasonic (Mansfield, TX, USA) was used as working electrodes. According to the manufacturer, these GS exhibit an electrical conductivity of 55.6 S cm^− 1^. A lab-made Ag|AgCl|KCl_(sat.)_ and a platinum wire were employed as reference and counter electrodes, respectively.

EIS measurements were performed at open circuit potential (+ 0.23 V *vs.* Ag|AgCl|KCl_(sat.)_) in the presence of 1 mmol L^− 1^ [Fe(CN)_6_]^3−/4−^ in 0.1 mol L^− 1^ KCl solution, applying an alternating potential with an amplitude of 10 mV in a frequency range from 50 kHz to 0.1 Hz. The equivalent Randles circuit was used to fit the experimental results and to determine the charge transfer resistance (*R*ct) related to the [Fe(CN)_6_]^3−/4−^ species.

The double-layer capacitance (*C*dl) was calculated by CV measurements ranging from 0.0 to + 0.30 V (vs. Ag|AgCl|KCl_(sat.)_) at different scan rates in 0.1 mol L^− 1^ KCl solution according to protocols from the literature [42]. The *C*dl value was obtained from the slope of the linear relationship between the current difference (Δ*I* = *I*a - *I*c) at + 0.15 V (normalized by the geometric area, A = 0.19 cm²) and the scan rate (5–30 mV s⁻¹). This potential, at which the current data were acquired, lies in the intermediate region of the selected potential window, where faradaic contributions are negligible and the measured current is dominated by double-layer charging, making it suitable for Cdl evaluation. Since *C*dl is directly proportional to the electroactive surface area (ECSA), the following Eq. (1) was used to estimate it, considering the specific capacitance value typically reported in the literature for graphitic materials (*C*s = 2.5 µF cm⁻²) [44] and the geometric area.

1 $$\:ECSA=\frac{Cdl\times\:A}{Cs}$$

Electron transfer kinetics at the electrode surfaces were evaluated by cyclic voltammetry using 1.0 mmol L⁻¹ [Ru(NH₃)₆]²⁺/³⁺ in 0.1 mol L⁻¹ KCl, with the scan rate varied from 10 to 200 mV s⁻¹. Under these conditions, the heterogeneous electron transfer rate constant (k^0^) can be estimated from the dependence of the peak-to-peak separation (Δ*E*p) on the scan rate. The Δ*E*p values obtained at each scan rate were used to calculate the corresponding dimensionless kinetic parameter (Ψ), enabling the determination of k^0^ by linear regression according to the method proposed by Lavagnini et al. [45] and Eqs. (2) and (3).

2 $$\:\varPsi\:=\:\frac{(-0.6288+0.0021{\Delta\:}E\mathrm{p})}{(1-0.017{\Delta\:}E\mathrm{p})}$$

3 $$\:\varPsi\:={k}^{0}{[\pi\:DnFv/RT]}^{-1/2}$$

Where D is the diffusion coefficient of [Ru(NH₃)₆]²⁺/³⁺, n is the number of electrons transferred in the electrochemical reaction, *R* = 8.314 J mol⁻¹ K⁻¹, T = 298 K, and F (Faraday’s constant) = 96,485 C mol⁻¹.

### Plasma treatment

In the proposed procedure, a custom-built robotic atmospheric air plasma system was employed, following an approach similar to that described by Di-Oliveira and colleagues [46], with minor adaptations for treating graphite sheet pieces cut into 1 × 1 cm squares.

Briefly, graphite sheet pieces are positioned on the motion platform, and the operator uses a micrometric adjustment stage to precisely control the needle-to-sample distance, ensuring uniform height alignment across all specimens. The touchscreen interface enables the user to define the treatment area by creating a program or designing a project that specifies the regions to be activated. It also allows the configuration of essential parameters, such as the spacing between successive plasma scan passes (with 0.1 mm precision) and the movement speed of the XY platform (adjustable from 50 to 300 mm min⁻¹). In addition, the interface supports the selection of different geometric patterns (square, rectangular, and elliptical), allowing greater flexibility according to the electrode geometry and experimental requirements.

The non-thermal (cold) atmospheric plasma is generated by a Tesla coil, in which a high voltage is applied between the plasma tip (anode) and the graphite sheet surface (cathode), promoting the ionization of atmospheric gases in the gap and producing the characteristic arc plasma. Plasma power is regulated through the control unit, which allows the fine adjustment of voltage and current to ensure stable and reproducible plasma generation. As the applied potential increases, a strong electric field is established, causing the dielectric breakdown of the surrounding gas and creating a conductive plasma channel through which current flows. This discharge produces the characteristic luminous emission of arc plasmas. A dielectric barrier at the generator tip contributes to the discharge stability, enabling localized and efficient treatment of the graphite surface.

The plasma treatment was conducted under optimized conditions, defined through univariate analysis, with an average duration of approximately 130 s per cm², allowing for precise, reproducible, and rapid surface activation of the graphite sheets. For all experiments, the scan line spacing was fixed at 0.2 mm, the scanning speed of the XY platform was set to 300 mm s⁻¹, the needle-to-sample working distance was maintained at 2.0 mm, and the plasma power was fixed at 11.4 W.

### Morphological and spectroscopic characterization

Raman spectra were recorded using a HORIBA LabRAM HR evolution spectrometer, equipped with an OSD Syncerity detector and a 532 nm wavelength laser. Scanning Electron Microscopy (SEM) images were acquired with a Tescan VEGA 3 LMU microscope, operating at 20 kV. Contact angle measurements for each electrode surface were carried out using a smartphone mounted on a universal holder. Images (*n* = 3) were captured 10 s after placing a drop (60 µL) of deionized water onto the working electrode surface. The contact angle was calculated on both left and right sides using GeoGebra^®^ software, and the average values were then estimated (https://www.geogebra.org/classic).

XPS measurements were conducted using an AXIS Supra system (Kratos, UK), equipped with a monochromatic Al Kα X-ray source (1486.6 eV) operating at 225 W and a hemispherical sector analyser. The instrument was set to fixed transmission mode, with a pass energy of 160 eV for survey scans and 20 eV for high-resolution scans. The collimator was configured in slot mode to probe an area of approximately 700 × 300 μm. At 20 eV pass energy, the full width at half maximum (FWHM) of the Ag 3d₅/₂ peak was 0.613 eV. The binding energy scale was calibrated by referencing the sp²-hybridized C1s peak at 284.5 eV, a standard calibration approach, albeit with known limitations [47], used here due to the absence of more reliable alternatives and because absolute binding energies were not the primary focus of the analysis.

## Results and discussion

### Optimization of robotic plasma treatment

The plasma processing parameters (scanning speed, distance between the needle and the GS surface (height), and the distance between lines) were systematically optimized using the CV response of a 1.0 mmol L⁻¹ [Fe(CN)_6_]^3−/4−^ redox couple in a 0.1 mol L⁻¹ KCl solution as the supporting electrolyte. Figures S1A-C show the optimization results for height (0.5–2.0 mm), line spacing (0.1–0.6 mm), and scanning speed (150–300 mm min^− 1^), respectively. As shown in Figures S1A-C, no significant changes were observed in the voltammetric profile of the redox probe when varying the distance between the needle and the GS surface and the scanning speed. These results indicate that the plasma treatment is highly precise within these ranges and that small variations do not significantly affect the performance of the proposed activation process. On the other hand, the optimization of line spacing revealed slight variations in the current response of the ferricyanide, likely due to a greater amount of graphite material being treated when shorter distances between lines were used. Additionally, the effect of performing the treatment with lines only in the horizontal direction and with horizontal followed by vertical lines was also evaluated, and no significant differences were observed (Figure S1D). Thus, plasma treatment was carried out under the following conditions: line spacing = 0.2 mm, scanning speed = 300 mm min^− 1^, and working distance = 2.0 mm. These parameters were carefully selected to ensure good repeatability and efficient processing speed during the treatment procedure.

After selecting the optimal parameters for the robotic air atmospheric plasma treatment system, the electrochemical activity of untreated and plasma-treated GS electrodes was evaluated using CV measurements with a 1.0 mmol L^− 1^ [Fe(CN)_6_]^3−/4−^ in a 0.1 mol L^− 1^ KCl solution (Fig. 2A). As observed, the untreated electrode exhibited a poorly defined voltammetric profile, characterized by a peak-to-peak separation of Δ*Ep* = 972 ± 3 mV and a low anodic peak current (~ 30 µA), indicating sluggish electron transfer kinetics and poor electrochemical reversibility in these conditions. In contrast, after the plasma treatment, the GS electrode exhibited a significantly improved electrochemical response, with a Δ*E*p of 96 ± 1 mV and a substantially higher anodic peak current (~ 70 µA - more than double the original value), indicating enhanced electron -transfer kinetics and markedly improved electrochemical reversibility.

This enhancement can be attributed to the energetic electron discharge generated during plasma treatment, which breaks the bonds between sp²-hybridized carbon atoms in the GS structure [33]. The resulting unsaturated carbon sites can react with reactive species produced from ionized atmospheric gases (OH^−^, O_2_^−^, etc.), leading to the formation of oxygen-containing functional groups on the electrode surface [34, 48, 49]. According to the literature [50], inner-sphere redox species such as ferricyanide can chemically interact with oxygen-containing functional groups on the GS surface, thereby facilitating electron transfer between the redox species and the electrode. It is important to mention that the resulting oxygen-containing functional groups improve interfacial charge transfer, without significantly affecting the bulk electrical conductivity of the GS, as reported in previous studies [31].

**Fig. 1:**
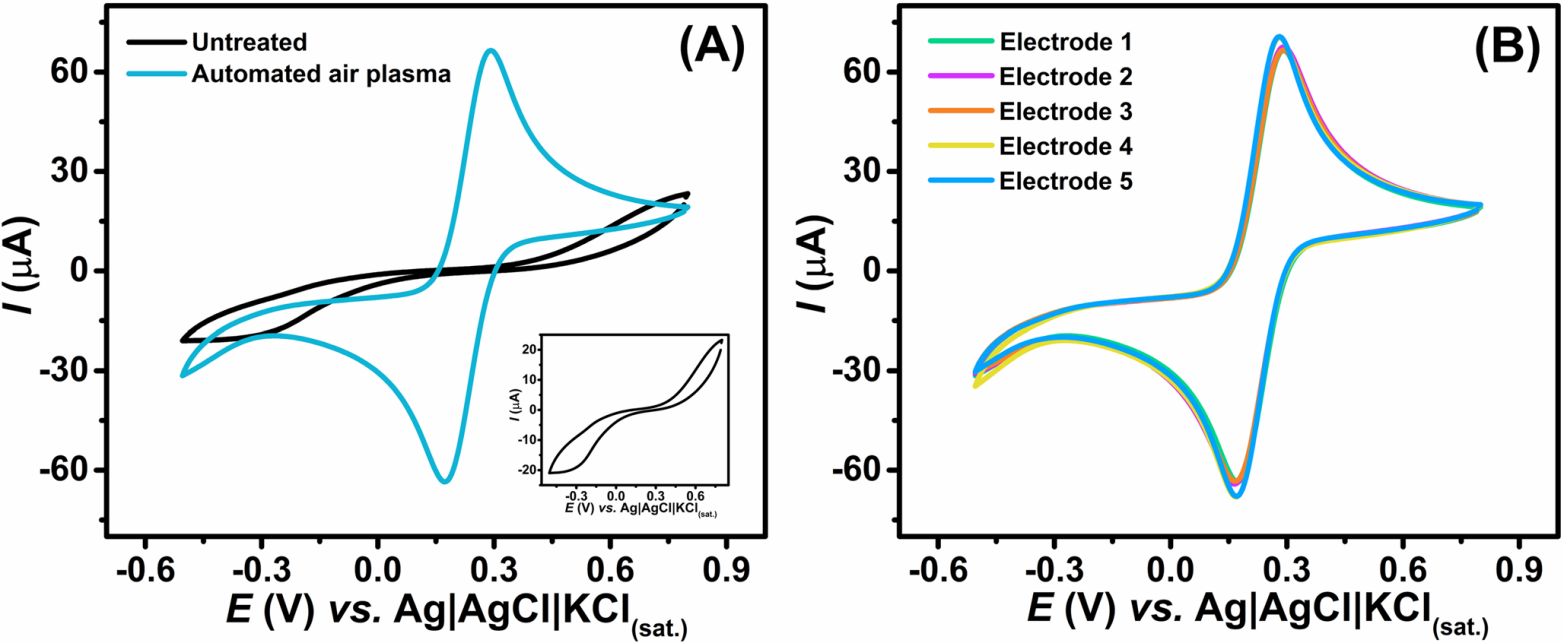
CVs recorded in the presence of: (**A**) 1.0 mmol L^− 1^ [Fe(CN)_6_]^3−/4−^ in 0.1 mol L^− 1^ KCl solution at 50 mV s^− 1^ before (black line) and after (blue line) robotic atmospheric plasma treatment; (**B**) CVs obtained for 1.0 mmol L^− 1^ [Fe(CN)_6_]^3−/4−^ in 0.1 mol L^− 1^ KCl solution at five independently prepared plasma-treated GS electrodes (*n* = 5)

The exfoliation of GS has been investigated by different studies in the literature [32, 51, 52]. However, the results obtained in the present work are notably superior to those reported in these previous studies, as evidenced by a significantly lower peak-to-peak separation (Δ*Ep* = 96 ± 1 mV at 50 mV s⁻¹) shown in Fig. 1A for the electrochemical response of 1.0 mmol L⁻¹ [Fe(CN)_6_]^3−/4−^. For example, Cai et al. employed Kapton tape to exfoliate graphite sheets and reported a peak-to-peak separation of 200 mV for ferricyanide at a scan rate of 50 mV s^− 1^ [51].

Furthermore, plasma treatment has previously been applied as a method for graphite exfoliation by our research group [32, 53]. For instance, Pereira et al. [53]. employed a conventional plasma-enhanced chemical vapor deposition (PECVD) process to exfoliate GSs, reporting a Δ*E*p of 114 mV at 50 mV s^− 1^ for the same redox couple. However, this method is associated with high equipment and operational costs. In another study, Marra and collaborators [32] used a handheld plasma pen to treat graphite paper, achieving a Δ*E*p of 116 mV. While this approach is more cost-effective, it requires longer treatment times and presents limited reproducibility due to its strong dependence on operator skills.

Notably, the inter-electrode precision achieved using robotic atmospheric plasma treatment was markedly superior. When evaluated using the CV profiles of 1.0 mmol L⁻¹ [Fe(CN)_6_]^3−/4−^ across five independently prepared electrodes (*n* = 5), the relative standard deviation (RSD) of the anodic peak current was below 5.7% (Fig. 1B). In contrast, the handheld plasma pen method reported in the literature resulted in an RSD greater than 18.1% when different operators were involved (Figure S2). These findings clearly demonstrate that robotic plasma treatment not only enhances electrochemical performance but also significantly improves reproducibility by minimizing operator-induced variability.

In addition to the excellent inter-electrode reproducibility, the long-term stability of the plasma-treated electrodes was evaluated over a period of 15 days. The anodic peak current for [Fe(CN)_6_]^3−/4−^ exhibited variations of less than 3% (data not shown), indicating that the electrode modification is highly stable over time and demonstrates excellent inter-day reproducibility.

To investigate the effects of plasma treatment on the GS surface, a comprehensive characterization was performed using Raman spectroscopy, atomic force microscopy (AFM), scanning electron microscopy (SEM) images, contact angle measurements, and electrochemical analysis. All characterizations were carried out both before and after plasma activation of the GS surfaces.

### Morphological and spectroscopic characterization

**Fig. 2:**
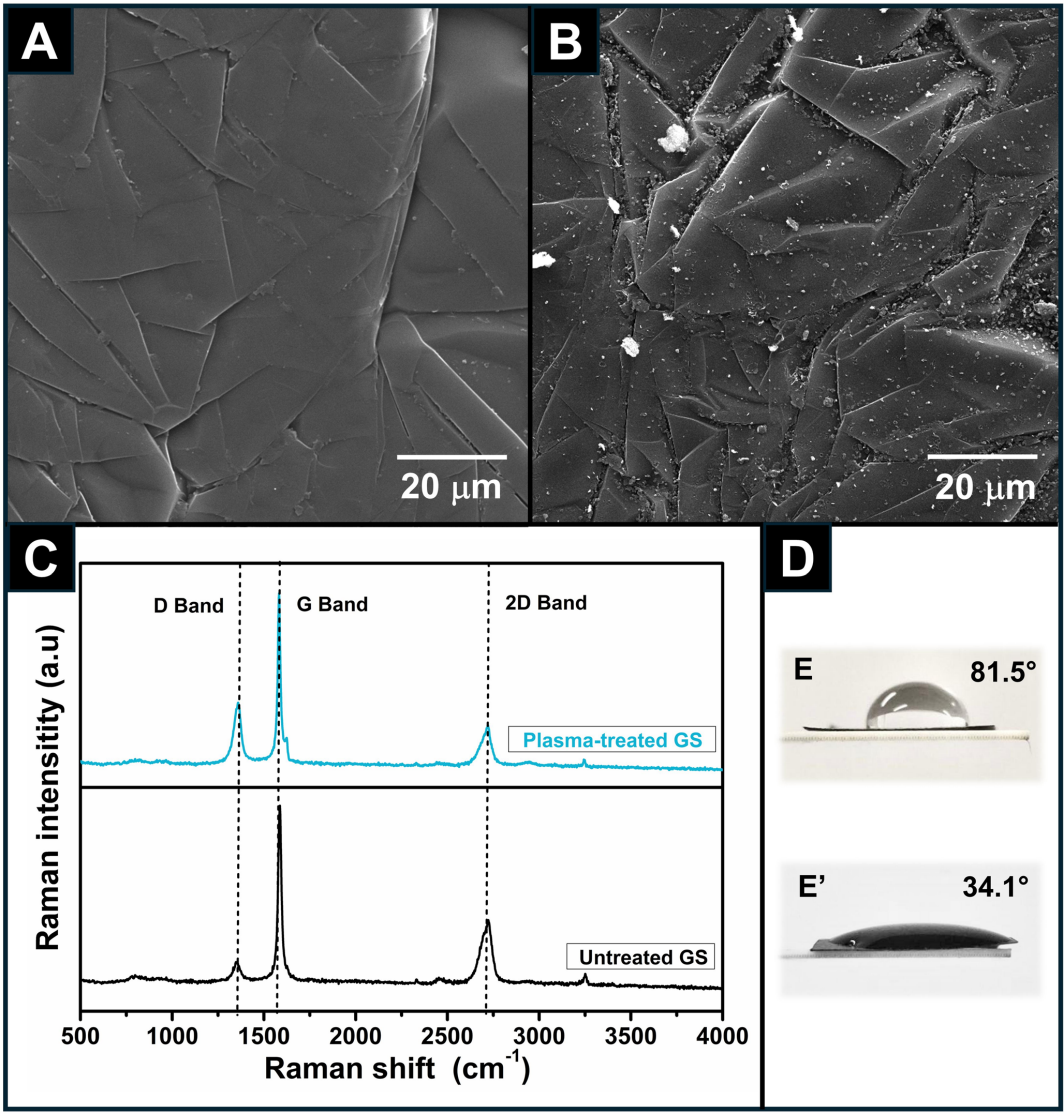
(**A**,** B**) SEM images of GS surfaces before and after robotic plasma treatment, respectively. (**C**) Raman spectra for untreated (black line) and plasma-treated (blue line) GS surfaces. (**D**) Contact angle measurements for (**E**) untreated and (**E’**) plasma-treated GS surfaces

The surface morphology of GS electrodes was analyzed by SEM images before and after plasma treatment. As shown in Fig. 2A, the untreated electrodes exhibited randomly stacked, micrometer-sized graphitic platelets with pronounced folds and wrinkles. This disordered structure is attributed to the GS fabrication process, which involves compacting thermally expanded graphite particles with relatively low density (approximately 1.11 g cm^− 3^) [25]. After plasma treatment (Fig. 2B), the GS surface displayed a more irregular morphology, characterized by the presence of nanoparticles, nanosheets, and nanoflakes, likely resulting from plasma-induced surface exfoliation.

These results are consistent with previous findings reported in the literature [32, 54], which describe that the most significant effects of plasma treatment are primarily associated with the oxygen content present in atmospheric air plasma. These authors explain that oxygen not only facilitates the removal of surface atoms and contaminants but also provides sufficient energy to penetrate the inner layers of the GS structure.

Raman spectroscopy was also conducted to investigate the effects of robotic plasma treatments on GS surfaces (Fig. 2C). The Raman spectra revealed three characteristic bands of graphitic materials. The D band (~ 1358 cm⁻¹) is associated with structural defects and disorder, particularly at the edges and within smaller sp^2^ carbon domains, often resulting from the incorporation of heteroatoms such as oxygen or nitrogen. The G band (~ 1571 cm⁻¹) corresponds to the in-plane vibrational modes of sp^2^- hybridized carbon atoms and is indicative of graphitic structural order. The 2D band (~ 2711 cm⁻¹) provides insights into the number of graphene layers and is highly sensitive to stacking order, structural disorder, and the presence of defects [55].

As evidenced by the Raman spectra, the D band intensity of the plasma-treated GS electrode is significantly higher than that of the untreated surface, indicating a successful introduction of additional structural defects in the graphitic lattice. These defects play a crucial role in enhancing the electrochemical properties of the material, as they facilitate charge transfer by reducing charge-transfer resistance and increasing the interaction with inner-sphere redox species, such as ferricyanide [52, 56]. Moreover, the intensity ratio of the D and G bands (I_D_/I_G_) provides a quantitative measure of structural disorder. Before plasma treatment, the I_D_/I_G_ ratio was 0.11; however, after treatment it increased to 0.38, confirming the formation of defects within the graphitic structure on the GS surface.

These results are consistent with previous studies showed that plasma treatment in the presence of atmospheric air can lead to the formation of reactive radicals capable of cleaving C = C bonds in carbon-based materials, thereby inducing structural defects [34, 54]. This process is often accompanied by a nanostructuring effect, in which the graphitic surface is fragmented into thinner nanosheets that later undergo partial restacking. However, during this restacking process, the nanosheets may not realign perfectly with the original crystalline structure, resulting in lattice distortions and an increased density of defects, as also observed by SEM images [32].

The intensity ratio between the 2D and G bands (I_2D_/I_G_) provides valuable insight into the structural characteristics of graphitic materials. Typically, I_2D_/I_G_ ratios greater than 1 are indicative of monolayer graphene [57–59]. In this study, the I_2D_/I_G_ ratios obtained before and after the robotic plasma treatment were 0.35 and 0.23, respectively. These values indicate that both samples consist of multilayer graphene. The decrease in the I_2D_/I_G_ ratio after plasma treatment does not suggest an increased degree of exfoliation but rather points to enhanced stacking of graphene layers and/or increased structural disorder affecting the 2D band. Such behavior is consistent with plasma-induced fragmentation of graphene sheets followed by partial restacking, leading to nanoscale structuring and a higher defect density, as also evidenced by the evolution of the D band [32, 52].

Water contact angle measurements were conducted to determine whether plasma treatment also affects the wettability of the GS surfaces (Fig. 2D). The water contact angle (θ), defined as the angle between the tangent to a hemispherical droplet at the point of contact and a line parallel to the surface, decreases with increasing surface hydrophilicity [60]. The activation or treatment of the carbon surface involves the incorporation of polar oxygen-containing functional groups, or surface etching, which enhances surface roughness and porosity by removing binders, impurities, and other non-conductive residues [49].

As observed, the contact angles for the untreated and plasma-treated GS electrodes were 85.1° and 34.1°, respectively. This pronounced reduction indicates that the GS surface became significantly more hydrophilic following plasma treatment. These contact angle measurements align with the Raman spectroscopy results, which confirmed the introduction of additional surface defects, particularly polar functional groups, that increase surface wettability.

**Fig. 3:**
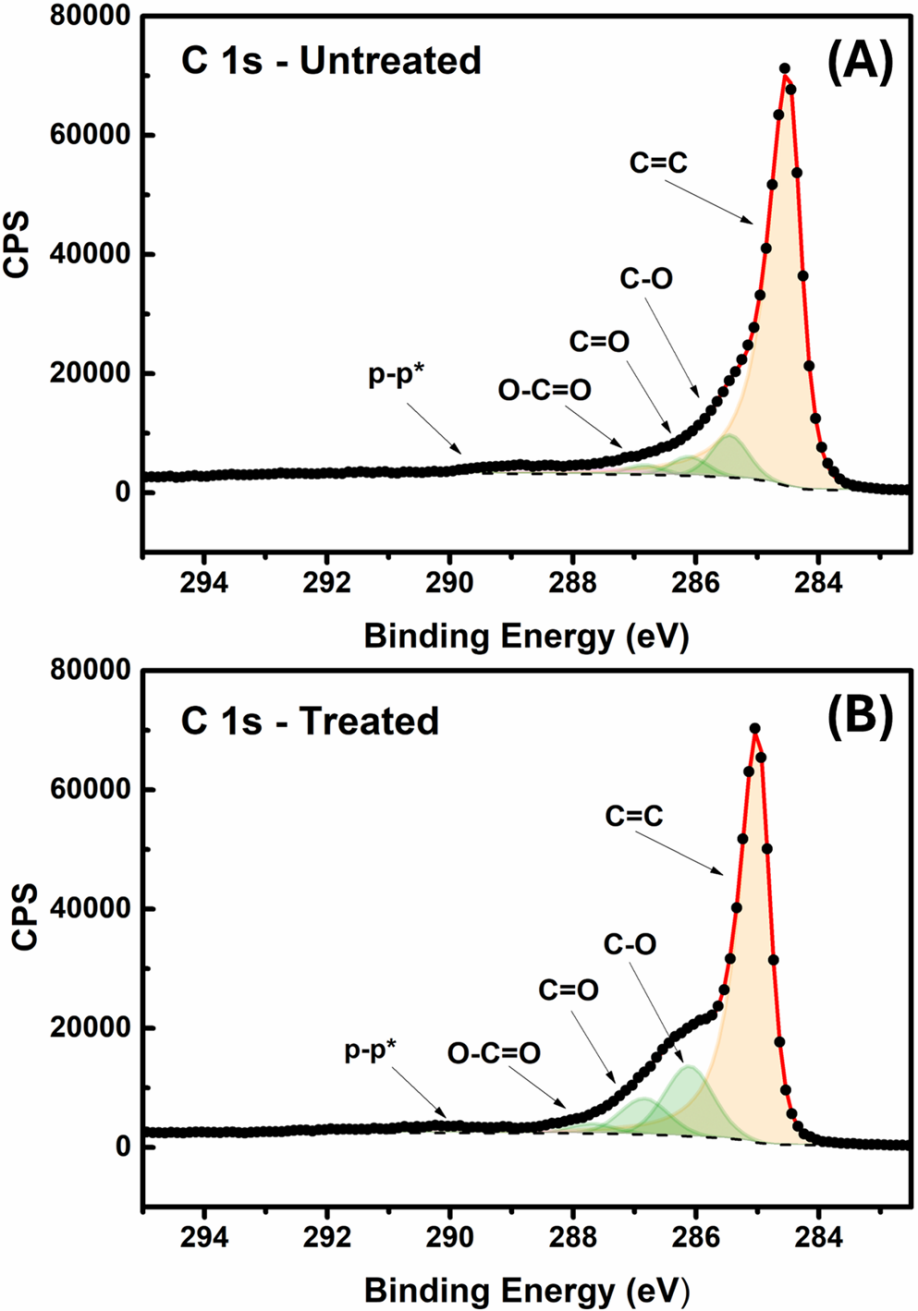
High-resolution deconvoluted C 1 s XPS spectra of the electrode surface before (**A**) and after (**B**) plasma treatment

XPS analysis was performed on both the untreated and plasma-treated GS electrodes, Fig. 3. In both cases a clearly defined asymmetric peak was observed at 284.5 eV, which is attributed to the sp^2^ hybridized graphitic bonding within graphite [61, 62]. Alongside this in both samples, three symmetric peaks were required for adequate fitting, corresponding to the C-O, C = O, and O-C = O bonding found within the samples. Importantly, following the plasma treatment process, an increase in the atomic concentrations of these carbon-oxygen moieties was found. In the untreated sample, these groups contributed to 7.6%, 3.4%, and 1.8%, respectively. Once treated with plasma activation, these values increased to 15.3%, 7.9%, and 2.2%, respectively, further confirming the generation of oxygen-containing functional groups on the surface of the graphite.

### Electrochemical detection of target analytes

**Fig. 4:**
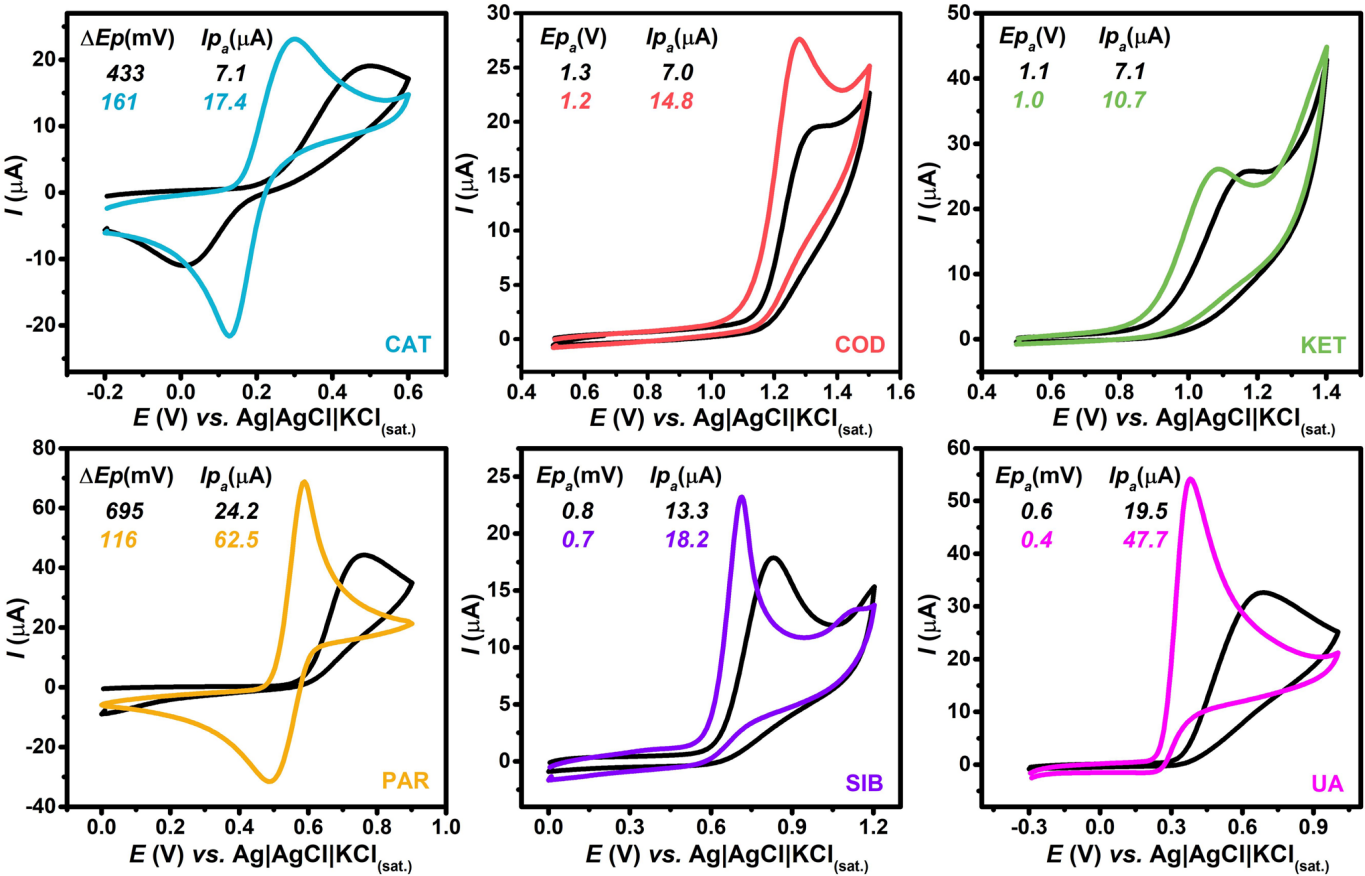
CV responses obtained for various compounds all at a concentration of 500 µmol L^− 1^, including: **(CAT)** catechol in BR buffer (pH 7.0), **(COD)** codeine in BR buffer (pH 2.0), **(KET)** ketamine in BR buffer (pH 9.0), **(PAR)** paracetamol in acetate buffer (pH 4.0), **(SIB)** sibutramine in BR buffer (pH 6.0) and **(UA)** uric acid in BR buffer (pH 7.0) using untreated and plasma-treated GS electrodes as working electrodes. **CV conditions**: scan rate = 50 mV s^− 1^; step potential = 5 mV

Initially, various compounds, including catechol, codeine, ketamine, paracetamol, sibutramine, and uric acid, each at a concentration of 500 µmol L^− 1^, were evaluated by CV at a scan rate of 50 mV s⁻¹, using both untreated and plasma-treated GS electrodes as the working electrode (Fig. 4). The untreated GS exhibited inferior electrochemical performance for all analytes evaluated. In contrast, plasma treatment resulted in a negative shift in peak potential across all analytes, accompanied by an increase in peak currents in most cases, indicating enhanced electrocatalytic activity. This improvement has been attributed in previous studies to chemical interactions between oxygen-containing functional groups on the GS surface and the analytes during the redox process [63, 64]. For instance, hydrogen bonding between surface oxygen-containing functional groups and the carbonyl oxygen of ascorbic acid enhances the acidity of the hydroxyl proton in its structure, thereby facilitating its oxidation [65].

Given the differences in the electrochemical responses observed for the organic compounds (Fig. 4) and the ferricyanide redox probe (Fig. 1) following robotic plasma treatment of the GS surface, we sought to investigate whether these changes could be attributed to an increase in ECSA. First, the *C*dl was calculated as described in the Experimental section (Figure S3). The estimated *C*dl values were 17.2 and 85.4 µF cm^− 2^ for the untreated and plasma-treated electrodes, respectively. Since, *C*dl is directly proportional to ECSA, and assuming a specific capacitance (*C*s) of 2.5 µF cm^− 2^ for graphitic-based materials [44] along with a geometric area (*A*) of 0.19 cm^2^, the estimated ECSA increased from 1.3 cm^2^ (untreated) to 6.5 cm^2^ (treated), representing a 5-fold enhancement. This finding is consistent with the SEM images, which revealed a highly irregular surface morphology. Moreover, cyclic voltammograms recorded in the presence of an outer-sphere redox probe (1.0 mmol L⁻¹ [Ru(NH_3_)_6_]^2+/3+^) showed an enhanced current response, which further corroborates the increase in the electrochemically active surface area (ECSA). This behavior can be explained by the fact that the ruthenium redox system acts as an outer-sphere probe, in which electron transfer occurs without the formation of specific chemical bonds or strong interactions with the electrode surface. As a result, its electrochemical response is less sensitive to surface defects, functional groups, or surface heterogeneity, thereby promoting faster electron transfer kinetics and well-defined redox peaks, even on untreated graphite surfaces.

EIS experiments were subsequently carried out to gain further insight into the surface properties of both GS electrodes. Figure S4 presents the Nyquist plots, which were fitted using a Randles equivalent circuit to reliably estimate the *R*ct. This model allows the evaluation of interfacial parameters such as the solution resistance (Rs), Rct, diffusion-related mass transport (Warburg impedance), and the *C*dl. To account for surface roughness and non-ideal capacitive behavior, *C*dl was replaced by a Constant Phase Element (CPE), which accounts for deviations from ideal behavior [66]. The calculated *R*ct values were 24.41 ± 0.90 kΩ for the untreated electrode and 0.31 ± 0.01 kΩ for the plasma-treated electrode, indicating a substantial enhancement in electron transfer kinetics because of the treatment.

CV scan rate studies were also performed in the presence of 1.0 mmol L^− 1^ [Ru(NH_3_)_6_]^2+/3+^ to determine the heterogeneous electron transfer rate constant (k⁰), using the method proposed by Lavagnini et al. [45]., as described in the Experimental section, in which the values of k^0^ were determined from the slope of the linear plot of Ψ vs. [πDnFv/RT]^−1/2^ according to Eqs. (2) and (3). The plasma-treated electrodes exhibited a significantly higher k⁰ value (1.8-fold greater) compared to untreated GS electrodes (Figure S5). This result is consistent with the improved electrochemical behavior observed in both EIS and CV responses for various analytes, confirming the effectiveness of the plasma treatment in promoting faster electron transfer processes. A comparative summary of the key electrochemical parameters obtained for the untreated and plasma-treated GS electrodes in the presence of [Ru(NH_3_)_6_]^2+/3+^ or [Fe(CN)_6_]^3−/4−^ is presented in Table 1. The results highlight the substantial improvements in electron transfer kinetics, surface activity, and overall electrochemical performance following plasma treatment.

**Table 1:** Comparison of key electrochemical parameters, including anodic (*I*p_a_) and cathodic (*I*p_c_) peak currents, peak-to-peak separations (Δ*E*p), heterogeneous electron transfer (k^0^), electrochemical surface area (ECSA), and charge transfer resistance (*R*ct) obtained for non-treated and plasma-treated GS electrodes in the presence of ^a^[Ru(NH_3_)_6_]^2+/3+^ or ^b^[Fe(CN)_6_]^3-/4^

Parameters	Untreated	Plasma treated
^*a^*I*p_a_ (µA)	21.3 ± 0.5	24.1 ± 0.4
^*a^*I*p_c_(µA)	20.4 ± 0.4	21.0 ± 0.3
^*b^*I*p_a_ (µA)	1.5 ± 0.1	71.3 ± 1.1
^*b^*I*p_c_ (µA)	5.1 ± 0.6	69.8 ± 2.8
^*a^Δ*E*p (mV)	73 ± 1	68 ± 1
^*b^Δ*E*p (mV)	972 ± 3	96 ± 1
^a^k^0^ (cm s^−1^)	(4.4 ± 0.2) ⋅ 10^− 3^	(8.0 ± 0.2) ⋅ 10^− 3^
ECSA (cm^2^)	1.3	6.5
^b^*R*ct (kΩ)	24.41 ± 0.90	0.31 ± 0.01

*CV experiments were conducted at 50 mV s^− 1^. These analyses were carried out in the presence of 1.0 mmol L^− 1^ ^a^[Ru(NH_3_)_6_]^2+/3+^ (Figure S6) or ^b^[Fe(CN)_6_]^3−/4−^ (Fig. 1) in 0.1 mol L^− 1^ KCl solution

### Electrochemical detection of uric acid

**Fig. 5:**
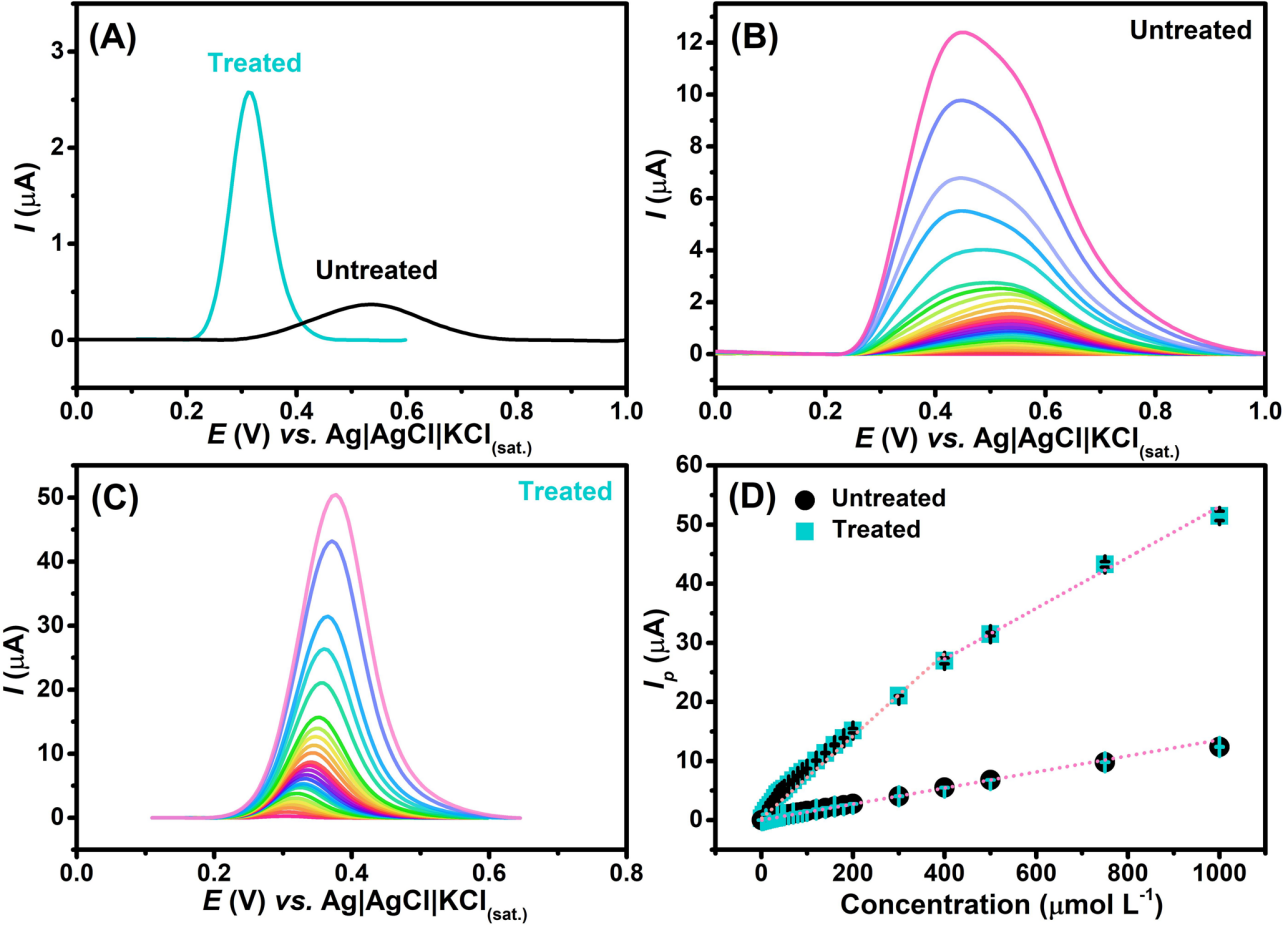
DPV scans recorded in the presence of (**A**) 10.0 µmol L^− 1^ UA at untreated (black line) and plasma-treated (blue line) GS electrodes; and for increasing UA concentrations (1.0 to 100.0 µmol L^− 1^) using GS electrodes (**B**) before and (**C**) after plasma treatment. (**D**) Comparison between calibration plots of non-treated and treated GS electrodes. All experiments were conducted using BR buffer (pH 7.0) as a supporting electrolyte. **DPV conditions**: *a* = 70 mV; *tm* = 50 ms and ΔEs = 6 mV

As proof of concept, the electrochemical detection of uric acid (UA), an important clinical biomarker, was investigated to demonstrate the applicability of the plasma-treated electrode. Firstly, the electrochemical detection of 10.0 µmol L⁻¹ UA was compared using DPV at both untreated and plasma-treated GS electrodes, with BR buffer (pH 7.0) as the supporting electrolyte, Fig. 5A. At the untreated electrode, a broad and poorly defined oxidation peak with lower current intensity was observed. In contrast, the plasma-treated electrode exhibited a significantly higher peak current, a shift of the oxidation potential toward more negative values, and a sharper, more symmetrical peak shape, indicating improved electrochemical reversibility and signal definition. As expected, this improved electrochemical performance is consistent with previous characterizations, which demonstrated an increase in the ECSA and a higher k^0^ value following the plasma treatment.

Afterwards, the influence of pH on the electrochemical response of 10.0 µmol L⁻¹ UA was examined using BR buffer as the supporting electrolyte over a pH range of 2.0 to 12.0, employing DPV and the plasma-treated GS electrode (Figure S6). As expected, the oxidation peak potential shifted toward more positive values with decreasing pH. Furthermore, the plot of peak potential versus pH exhibited a slope of 59 mV pH⁻¹, which is consistent with the Nernstian behavior described by the Nernst equation [67]. This result suggests a proton-coupled electron transfer process involving an equal number of protons and electrons [68].

The electrochemical oxidation mechanism of UA has been extensively studied and is well established in the literature. It is generally accepted that the oxidation process involves the transfer of two protons and two electrons, leading to the formation of a diimine intermediate. This species is highly unstable in aqueous solution and undergoes rapid hydrolysis to produce an imine-alcohol derivative [68–70].

The highest peak current was observed at pH 7; therefore, this condition was selected as the optimal medium and used as the supporting electrolyte in subsequent experiments. CV scan rate studies were then carried out in the range of 10 to 200 mV s⁻¹ to investigate its influence on the electrochemical behavior of 500 µmol L⁻¹ UA in BR buffer (pH 7.0), as shown in Figure S7. The results revealed that the electrochemical oxidation of UA is governed by a diffusion-controlled process, as evidenced by the linear relationship between peak current (*Ip*) and the square root of the scan rate (ν¹ᐟ²), with a correlation coefficient of *r* = 0.999. This finding was further supported by the plot obtained between log *Ip* and log *ν*, which yielded a slope of 0.591, consistent with a diffusion-controlled mechanism.

In the next step, DPV parameters were individually optimized by evaluating the peak, peak current intensity, and peak width at half-height, using 10.0 µmol L⁻¹ UA in BR buffer (pH 7.0). Figures S8 display the DPV scans obtained during the optimization of modulation amplitude (*a* = 10–100 mV), modulation time (*tm =* 10–100 ms), and step potential (ΔEs = 1–10 mV). The DPV parameters that provided the most favorable peak characteristics were *a* = 70 mV, *tm* **=** 50 ms, and ΔEs = 6 mV.

Under optimized conditions, calibration curves were obtained for both untreated (Fig. 5B) and plasma-treated (Fig. 5C) GS electrodes. A comparison between the resulting calibration plots is presented in Fig. 5D. Additionally, the analytical performance of each electrode is summarized in Table 2, which compares key parameters including the linear range, limit of detection (LOD), and sensitivity (slope). The LOD was estimated based on IUPAC definition (3σ/s), where σ is the standard deviation of linear coefficient obtained through the analytical curve and *s* is the analytical sensitivity of the linear curve (slope).

**Table 2:** Analytical parameters acquired for GS electrodes before and after plasma treatment for UA detection

Analytical Parameters	Untreated	Plasma Treated
Linear range (µmol L^−1^)	5.0–1000.0	1.0–400.0 and 400.0–1000.0
r	0.997	0.999
Intercept (µA)	−0.0114 ± 0.0041	0.1557 ± 0.0033
Slope (µA L µmol^−1^)	0.0168 ± 0.0003	0.1230 ± 0.0034
LOD (µmol L^−1^)	0.71	0.08

As observed, a significant enhancement in sensitivity, approximately 7-fold, was achieved following plasma treatment. In addition, the broader peak width recorded at the untreated electrode can hinder the ability to discriminate UA from other interfering analytes, thereby reducing overall selectivity. The lower LOD value achieved using plasma-treated GS electrodes demonstrates the suitability of this platform for the determination of UA in biological samples, as it allows reliable detection within the physiological concentration ranges reported for blood serum and urine [71, 72].

In addition to the increase in sensitivity, the oxidation potential of uric acid shifts toward less positive values, indicating enhanced electrocatalytic activity. This behavior is attributed to the plasma-induced increase in surface oxygen content and the introduction of oxygen-containing functional groups, which enhance the interaction between uric acid molecules and the electrode surface through hydrogen bonding and electrostatic interactions. These interactions promote more favorable adsorption and facilitate electron transfer, thereby reducing the overpotential required for oxidation. Similar effects have been reported in previous studies, where chemical interactions between oxygen-containing functional groups on graphitic surfaces and the analyte were shown to play a key role during the redox process [63].

The intra-electrode precision of the proposed method was evaluated through successive DPV measurements (*n* = 10) performed in the presence of 10.0 µmol L⁻¹ UA in BR buffer (pH 7.0) (Figure S9). The low relative standard deviation (RSD < 3.2%) confirmed the high repeatability of the proposed method at a single electrode. Additionally, inter-electrode precision was assessed using independently prepared plasma-treated GS electrodes (*n* = 3), yielding an RSD of approximately 4.7%, which indicates good reproducibility of the GS material and surface treatment process.

**Fig. 6:**
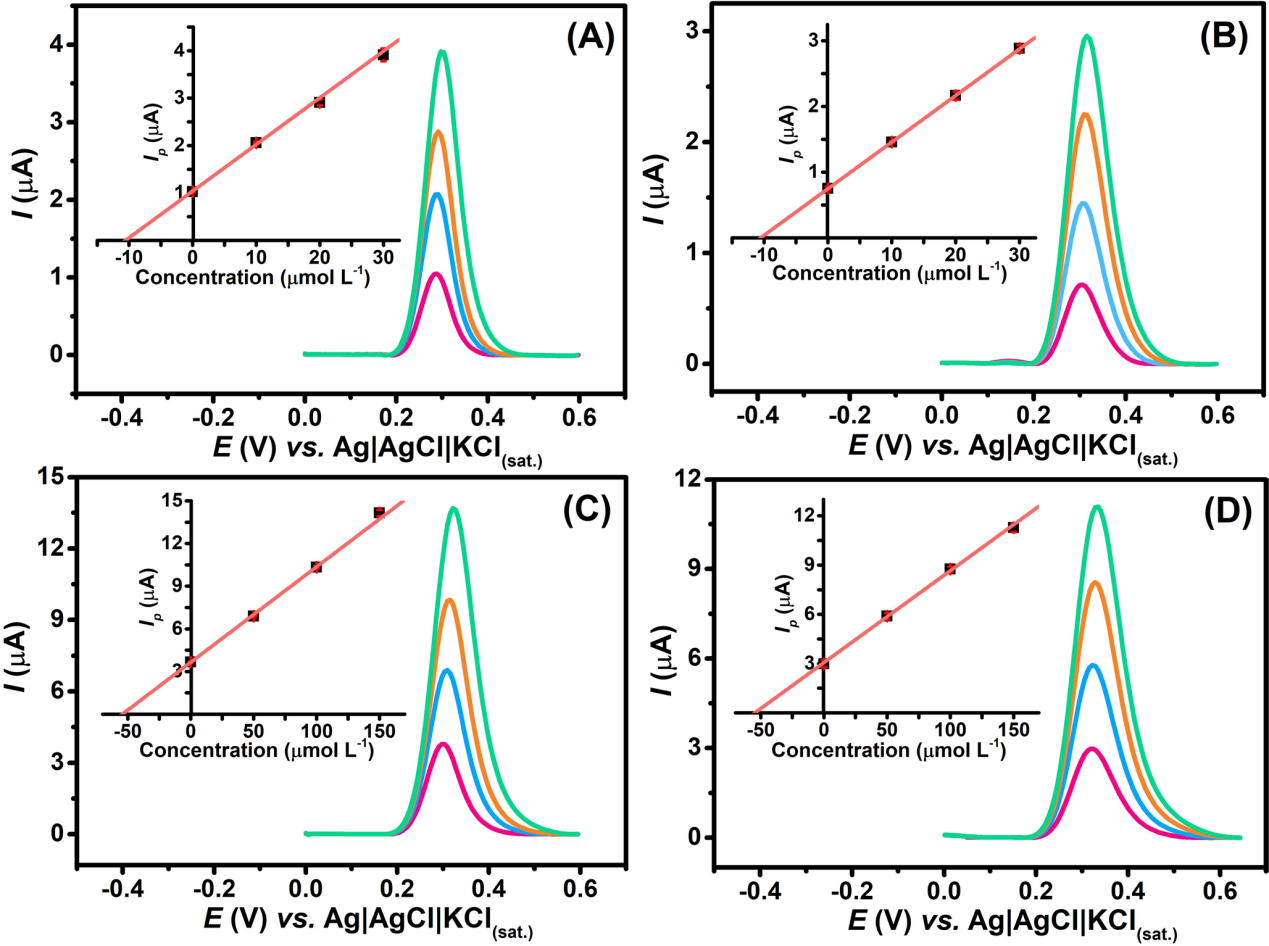
DPV scans obtained for the determination of UA in (**A**,** C**) synthetic saliva and (**B**,** D**) synthetic urine samples in two concentration levels (10 and 50 µmol L^− 1^). The inserted Figure corresponds to the calibration plots constructed using the standard addition method. **DPV conditions**: *a* = 70 mV, *tm* = 50 ms and ΔEs = 6 mV

To further demonstrate the practical applicability of the method, UA was determined in spiked synthetic saliva and urine samples using the standard addition method (see Table 2; Fig. 6). For each spiking level (10 and 50 µmol L^− 1^), three successive standard additions were performed. The recovery values obtained (106–109%) confirmed the method’s accuracy and reliability for UA quantification in complex biological matrices.

**Table 3:** Recovery values obtained for the analysis of synthetic saliva and synthetic urine samples spiked with two concentration levels of UA

Samples	Spiked (µmol L^− 1^)	Found (µmol L^− 1^)	Recovery (%)
Synthetic Saliva	10	10.6 ± 0.1	106 ± 1
	50	54.4 ± 1.0	109 ± 2
Synthetic Urine	10	10.6 ± 0.7	106 ± 7
	50	54.6 ± 1.9	109 ± 3

## Conclusions

In this work, we demonstrate that a low-cost, lab-made, and fully automated plasma treatment system, constructed using a simple Tesla coil commonly employed in educational and hobbyist electronics, can be effectively used for the rapid and reproducible activation of graphite sheet (GS) electrodes. This approach enhances their electrochemical performance through a scalable, straightforward, and operator-independent process. Key operational parameters of the plasma system constructed from a Tesla Coil Speaker, such as treatment height, line spacing, and scanning speed, were systematically optimized to ensure high reproducibility and consistent electrochemical responses. The resulting plasma-treated GS electrodes exhibited significantly enhanced performance compared to untreated ones, primarily due to the increased electroactive surface area resulting from plasma-induced graphite exfoliation through cyclic voltammetry with ferricyanide as a redox probe, key operational parameters—such as height, line spacing, and scanning speed were optimized to ensure high reproducibility and consistent electrochemical responses. The plasma-treated GS electrodes exhibited significantly improved performance compared to untreated ones, primarily due to an increase in electroactive surface area resulting from graphite exfoliation induced by the plasma process.

The effects of the plasma treatment on the graphite sheet (GS) surface were clearly evidenced by a combination of morphological, spectroscopic, and electrochemical characterizations. SEM imaging revealed the formation of 3D nanostructures and a pronounced increase in surface roughness—hallmarks of surface exfoliation and restructuring induced by the plasma process. These features were further supported by Raman spectroscopy, which showed a significant increase in the density of structural defects, indicating successful disruption of the graphitic order. Contact angle measurements corroborate the chemical activation of the surface, revealing enhanced hydrophilicity attributed to the incorporation of polar oxygen-containing functional groups. Complementary electrochemical analyses, including electrochemical impedance spectroscopy (EIS) and estimation of the heterogeneous electron transfer rate constant (k⁰), confirmed a substantial improvement in electron transfer kinetics—highlighting the effectiveness of the plasma treatment in producing highly active and responsive electrode surfaces. As a proof of concept, the developed platform was applied to the electrochemical determination of uric acid (UA) using differential pulse voltammetry (DPV) at both untreated and plasma-treated GS electrodes. A remarkable enhancement in analytical performance was observed, with the sensitivity increasing from 0.0168 ± 0.0003 µA L µmol⁻¹ to 0.1230 ± 0.0034 µA L µmol⁻¹ after the plasma treatment-representing a more than sevenfold improvement. These results demonstrate the potential of the proposed approach as a robust, reproducible, and operator-independent strategy for electrode activation, offering excellent analytical performance and minimized variability due to manual processing. As proof of concept, the determination of uric acid was performed by DPV scans, using both GS surfaces. The sensitivity (slope) enhanced from 0.0168 ± 0.0003 µA L µmol^− 1^ to 0.1230 ± 0.0034 µA L µmol^− 1^ after the treatment. This approach presents a promising route to treat electrodes, with excellent performance and reproducibility, minimizing human error.

## Supplementary Information

Below is the link to the electronic supplementary material.

Supplementary Material 1

## Data Availability

We added a statement in the text.
