# Supplementary material for: Automated air plasma-assisted functionalization of graphite electrodes for enhanced electrochemical sensing of uric acid
Source: Mikrochim Acta. 2026 Feb 28;193(3):199. doi: 10.1007/s00604-026-07939-2 (PMC12950079; doi:10.1007/s00604-026-07939-2)
Supplement: Supplementary file 1 — Supplementary Material 1 [file 604_2026_7939_MOESM1_ESM.docx]

**Automated Air Plasma-Assisted Functionalization of Graphite Electrodes for Enhanced Electrochemical Sensing of Uric Acid**

Mariana C. Marra^1^, Marina Di-Oliveira^1^, Raquel G. Rocha^1^, Teodoro R. Terra^1^, Robert D. Crapnell^2^, Craig E. Banks^2^, Eduardo M. Richter^1*^, Rodrigo A. A. Muñoz^1**^

*^1^Institute of Chemistry, Federal University of Uberlândia, 38408-100, Uberlândia, Brazil*

*^2^Faculty of Science and Engineering, Manchester Metropolitan University, Dalton Building, Chester Street, Manchester, M1 5GD, Great Britain*

**Corresponding author:**

*emrichter@ufu.br

**munoz@ufu.br


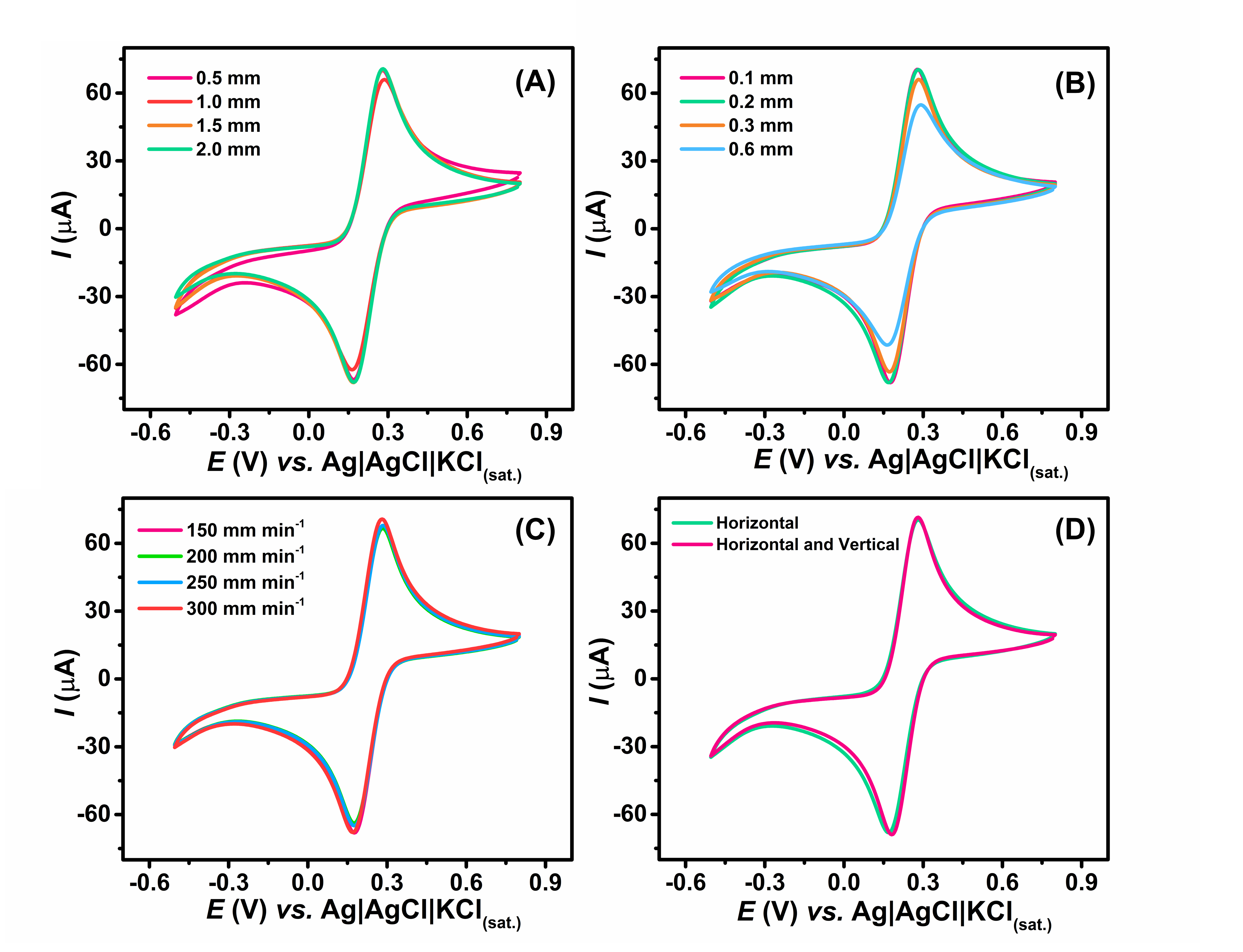


**Figure S1.** Optimization of plasma treatment parameters for GS electrodes, evaluated using cyclic voltammetry in 1.0 mmol L⁻¹ [Fe(CN)₆]³⁻/⁴⁻ solution in 0.1 mol L⁻¹ KCl. **(A)** Effect of distance between the plasma and GS surface (0.5–2.0 mm); **(B)** Effect of line spacing between successive plasma passes (0.1–0.6 mm); **(C)** Effect of scanning speed (150–300 mm min⁻¹); **(D)** Effect of treatment direction (horizontal vs. combined horizontal and vertical). **CV conditions:** scan rate = 50 mV s^-1^; step potential = 5 mV.





**Figure S2.** Cyclic voltammetric reproducibility of GS electrodes treated using a handheld plasma device. Five independently prepared electrodes (n = 5) were evaluated using 1.0 mmol L⁻¹ [Fe(CN)₆]³⁻/⁴⁻ solution in 0.1 mol L⁻¹ KCl. **CV conditions:** scan rate = 50 mV s^-1^; step potential = 5 mV.





**Figure S3.** Cyclic voltammograms recorded at scan rates ranging from 10 to 30 mV s^-1^ for **(A)** untreated and **(B)** plasma-treated GS electrodes. CVs were performed in 0.1 mol L^-1^ KCl solution from 0.0 to +0.3 V *vs.* (Ag|AgCl|KCl_(sat.)_). **(C)** Capacitance data: plots of Δ*j* (peak currents were measured at +0.15 V (*vs.* Ag|AgCl|KCl_(sat.)_) normalized by the geometric area. **CV conditions:** step potential = 5 mV.





**Figure S4.** Electrochemical impedance spectroscopy (EIS) Nyquist plots of GS electrodes in 0.1 mol L⁻¹ KCl solution, with a potential of applying +0.23 V *vs.* Ag|AgCl|KCl_(sat.)_, for untreated (black line) and plasma-treated (blue line) GS electrodes.





**Figure S5.** Effect of scan-rate (10−200 mV s^-1^) on the electrochemical response of 1.0 mmol L^-1^ [Ru(NH_3_)^6^]^2+/3+^ in 0.1 mol L^-1^ KCl solution, using **(A)** untreated and **(B)** plasma-treated GS as working electrodes. **CV conditions:** step potential = 5 mV.





**Figure S6. (A)** DPV responses recorded in the presence of 10.0 μmol L^−1^ UA in BR buffer at different pHs (2.0-12.0). **(B)** Plots of peak potential (*Ep*) and peak current (*Ip*) as a function of pH for the UA oxidation process. **DPV conditions:** *a* = 50 mV; *tm* = 50 ms and ΔEs = 5 mV.

**

**

**Figure S7. (A)** Cyclic voltammograms recorded at scan rates from 10 to 200 mV s^-1^ using the plasma-treated GS electrode. Measurements were performed in the presence of 500 μmol L^-1^ UA in BR buffer (pH 7.0). Plot of peak current (*Ip*) *vs* **(B)** scan rate or **(C)** square root of scan rate (v^1/2^) **(D)** log *Ip* *vs* log ν. **CV conditions:** step potential = 5 mV.

**

**

**Figure S8.** Baseline-corrected DPV responses for 10.0 µmol L⁻¹ UA at plasma-treated GS electrodes under different experimental conditions. (A, B) Effect of pulse amplitude (10–100 mV); (C, D) effect of modulation time (10–100 ms); (E, F) effect of step potential (1–10 mV). Panels A, C, and E show representative voltammograms, while panels B, D, and F present the corresponding peak current or peak width at half-height as a function of the studied parameter. **Supporting electrolyte:** BR buffer (pH 7.0).

**

**

**Figure S9. (A)** DPV scans obtained from ten successive measurements (n = 10) of 10.0 µmol L^-1^ AU using plasma-treated GS electrodes in BR buffer (pH = 7.0). **(B)** Corresponding bar plot of anodic peak currents (*Iₚ*). **DPV conditions:** *a* = 70 mV; *tm* = 50 ms and ΔEs = 6 mV.
